# Traumatic Bilateral Pneumothoraces due to Sternal Wire Migration

**DOI:** 10.1155/2012/438429

**Published:** 2012-05-27

**Authors:** Umar Imran Hamid, Scott Gillespie, Colum Lynchehaun, Haralabos Parissis

**Affiliations:** ^1^Department of Cardiothoracic Surgery, Royal Victoria Hospital, Belfast BT12 6BA, UK; ^2^Department of Radiology, Royal Victoria Hospital, Belfast BT12 6BA, UK

## Abstract

Sternal wound complications after cardiac surgery are associated with increased morbidity and mortality. Wire migrations associated with sternal dehiscence can lead to catastrophic haemorrhage unless intervened in time. We present a case of sternal wire migration causing bilateral pneumothoraces.

## 1. Introduction

Sternal wound complications, including dehiscence and infection, occur in 1% to 3% of patients undergoing cardiac surgery. These complications require extended hospitalization, long-term antibiotics and multiple operative procedures, resulting in high financial impact. Various factors affect the risk of sternal wound dehiscence ranging from patient comorbidities to operative techniques. Migrating wires resulting from sternal dehiscence present a clinical challenge and are source of mortality and morbidity.

## 2. Case History

A 67-year-old male presented with a ten-year history of chronic stable angina. Past medical history of significance included coronary artery stenting, bilateral stripping of varicose veins, ulcerative colitis, and previous history of smoking. Coronary angiogram revealed severe left main stem with three vessel disease and preserved left ventricular function. His current medication included aspirin, perindopril, atorvastatin, isosorbide mononitrate, bisoprolol, and azathioprine (for ulcerative colitis). He underwent coronary artery bypass grafts × 3 with left internal mammary artery to left anterior descending artery, right internal mammary artery through the transverse sinus to the marginal branch of the circumflex, and saphenous vein graft to the distal right coronary artery. The immediate postoperative period was uneventful.

**Figure 1:**
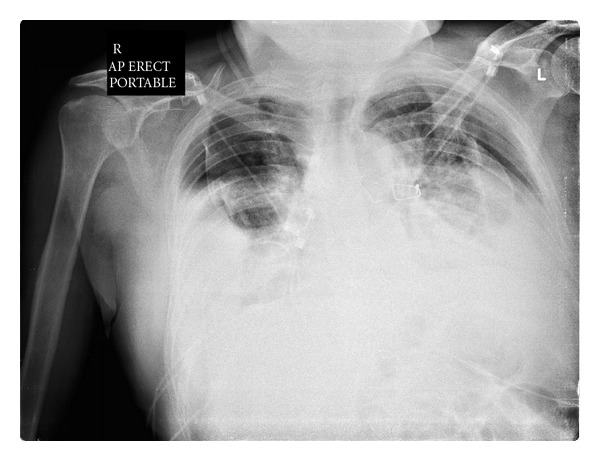
CXR showing bilateral pneumothoraces with displaced sternal wires.

**Figure 2:**
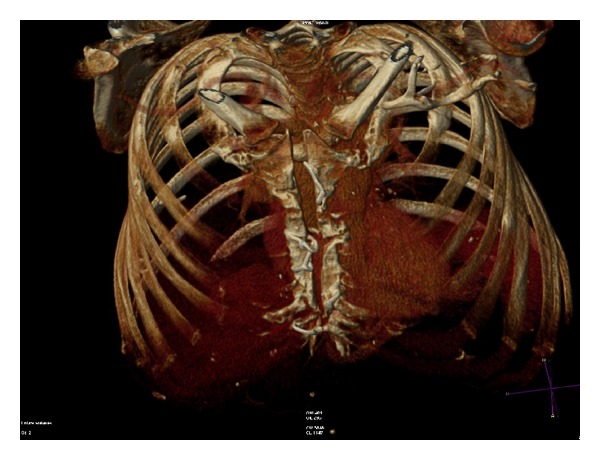
CT reconstruction of sternal dehiscence.

He presented 4 weeks later with dry cough and signs of sternal malunion; the sternum was unstable but the overlying skin was intact. Conservative approach with external thoracic support was initially used. He was readmitted two weeks later with persistent cough and acute shortness of breath. Chest roentegram showed laterally displaced sternal wires and bilateral hydro-pneumothoraces (Figure 1). Computed tomography (CT) scan was carried out which confirmed sternal dehiscence (Figure 2).

**Figure 3:**
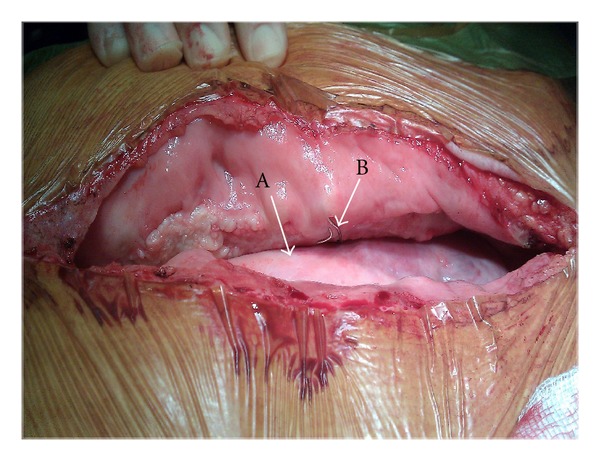
Intraoperative pictures of sternal dehiscence with Lung parenchyma (Arrow A) coming in contact with sternal wire (Arrow B).

He subsequently underwent sternal reconstruction. Intraoperatively, the sternum had completely dehisced; an upper left wire and a middle right wire (Figure 3) were evident protruding into the respective pleural cavities, coming in to contact with lung parenchyma. A third lower wire was confined between the inferior wall of the right ventricle and the diaphragm, to the right of the midline. After removal of these wires, the sternal edges were refashioned, pectoral muscles mobilised, and Robischek repair was carried out. Bilateral pleural, mediastinal, and subcutaneous drains were placed. The postoperative period was uneventful. At sixth-week followup, his sternum is healing well.

## 3. Discussion

Sternal wound complications after median sternotomy are infrequent and can be potentially devastating leading to morbidity and mortality [1].

Obesity, female sex, diabetic disease, bilateral internal mammary artery harvesting, postoperative renal failure, and prolonged ventilation are known factors that increase the risk of sternal complications [2, 3]. Harvesting of internal mammary artery (IMA) for coronary artery bypass grafting (CABG) can lead to decreased circulation to the sternum [4]. Caution should be exercised in patients on immunosuppressive drugs such as steroids and azathioprine who already have impaired wound healing while considering bilateral internal mammary artery harvesting.

Pain is the most frequent symptom encountered with sternal malunion. Some patients may be incapacitated by discomfort associated with fractured wires or nonunion. Others tolerate varying degrees of discomfort to avoid further operation.

Sternal wire migration is usually treated conservatively unless complications occur. Various complications of sternal wire migration have been published in the literature, including tamponade resulting from right ventricular injury [5], aortic injury [6], hemoptysis [7], and pulmonary valve endocarditis [8]. Right Ventricular laceration due to sternal wire migration is associated with mortality of up to 50%.

Migrated sternal wires should be assessed by radiological imaging to ascertain the potential risk of mediastinal injury. CT angiography allows precise and reproducible localization of vital mediastinal structures. When planning sternal reconstruction, CT scan can give important information concerning exact spatial location of conduits and proximity of cardiac chambers to the sternum, as well as location of sternal wires.

Bilateral pneumothoraces, on a background of sternal wire displacement, prompted us to proceed with urgent surgery. Radiological and intraoperative findings were consistent with sternal dehiscence and displaced sternal wires in proximity to lung parenchyma. The wires, on a background of persistent dry cough, could have account for direct lung injury with subsequent traumatic pneumothoraces.

In conclusion, sternal wire migration can be associated with fatal mediastinal injury. The intraoperative and postoperative management should be individualized to minimize sternal complications. Patients who have migrated wires should have radiological assessment to determine proximity to vital structures and be managed accordingly.
